# Easy Method of Extracting Eoreign Bodies from the Eye-Lids

**Published:** 1859-10

**Authors:** 


EASY METHOD OF EXTRACTING FOREIGN BODIES FROM THE EYE-LIDS.

We give insertion to the suggestions of Dr. LEON RENARD, as to the removal of foreign bodies from the conjunctiva, not certainly because of any novelty in the mode of procedure, for a similar course is pursued, and has been from time immemorial, by those wholly unacquainted with the anatomy of the eye and its appendages, but because of the ready explanation given of the *modus operandi*—as it is highly important that we Doctors should have a reason to give for the faith that is in us:

"Dr. Leon Renard, in a note to the Editor of the Union Medicale, describes the following method of extracting small sabstances which have become lodged in the groove formed by the reflection of the conjunctiva from the upper lid to the sclerotic, and which often cannot be seen, even when the lid is inverted. The lid being seized at its angles between the thumb and forefinger of each hand, is to be gently drawn forward and downward, as far as possible, over the lower lid, and retained there for about a minute. On allowing the upper lid to return to its normal position, the flow of tears will carry off the foreign body, which will usually be found on the lower lid, or one of the lashes, or on the cheek. The writer states that he has often found this simple method of the greathst utility and convenience."—*Char. Med. Jour. & Rev.*

